# Clinical Neuropathology practice news 1-2014: Pyrosequencing meets clinical and analytical performance criteria for routine testing of MGMT promoter methylation status in glioblastoma 

**DOI:** 10.5414/NP300730

**Published:** 2013-12-20

**Authors:** Matthias Preusser, Anna S. Berghoff, Claudia Manzl, Martin Filipits, Andreas Weinhäusel, Walter Pulverer, Karin Dieckmann, Georg Widhalm, Adelheid Wöhrer, Engelbert Knosp, Christine Marosi, Johannes A. Hainfellner

**Affiliations:** 1 Department of Medicine I, Medical University of Vienna, Vienna,; 2 Division of Pathology, Medical University of Innsbruck, Innsbruck,; 3 Comprehensive Cancer Center – CNS Tumors Unit (CCC-CNS), Medical University of Vienna, Vienna,; 4 Molecular Diagnostics, Austrian Institute of Technology,; 5 Department of Radiotherapy,; 6 Department of Neurosurgery, and; 7 Institute of Neurology, Medical University of Vienna, Vienna, Austria

**Keywords:** glioblastoma, MGMT, methylation, predictive, prognostic

## Abstract

Testing of the MGMT promoter methylation status in glioblastoma is relevant for clinical decision making and research applications. Two recent and independent phase III therapy trials confirmed a prognostic and predictive value of the MGMT promoter methylation status in elderly glioblastoma patients. Several methods for MGMT promoter methylation testing have been proposed, but seem to be of limited test reliability. Therefore, and also due to feasibility reasons, translation of MGMT methylation testing into routine use has been protracted so far. Pyrosequencing after prior DNA bisulfite modification has emerged as a reliable, accurate, fast and easy-to-use method for MGMT promoter methylation testing in tumor tissues (including formalin-fixed and paraffin-embedded samples). We performed an intra- and inter-laboratory ring trial which demonstrates a high analytical performance of this technique. Thus, pyrosequencing-based assessment of MGMT promoter methylation status in glioblastoma meets the criteria of high analytical test performance and can be recommended for clinical application, provided that strict quality control is performed. Our article summarizes clinical indications, practical instructions and open issues for MGMT promoter methylation testing in glioblastoma using pyrosequencing.

## Background 

Glioblastoma is the most frequent primary brain tumor of adults and is highly malignant with a median overall survival time of only 12 – 14 months [1]. The standard therapy options include maximal safe neurosurgical resection, radiotherapy and chemotherapy preferentially with the alkylating drug temozolomide, antiangiogenic treatment with the antibody bevacizumab, symptom control through corticosteroids for increased intracranial pressure and anticonvulsants and best supportive care [1, 2, 3, 4]. Important clinical prognostic factors comprise patient age at diagnosis, patient performance status, and the extent of resection [5]. At the molecular level, the O6-methylguanine-methyltransferase (MGMT) promoter methylation status carries relevant prognostic and, at least in the subset of elderly patients, also predictive information for therapy planning [6, 7, 8]. 

MGMT is a DNA-repair protein that counteracts the effect of alkylating chemotherapy by removing methyl groups from the O6-position of guanine. In line with this assumption, the MGMT promoter methylation status influences the outcome of patients with glioblastoma treated with temozolomide. In 2005, Hegi et al. [6] demonstrated in a post hoc analysis of a study cohort from a phase III study that glioblastoma patients aged 18 – 70 years with intratumoral MGMT gene silencing by promoter hypermethylation had significantly better outcomes when treated with combined radiochemotherapy with temozolomide as compared to patients treated with radiotherapy alone. However, these prognostic data did not clearly support withholding temozolomide from patients with unmethylated MGMT promoter in the absence of effective treatment alternatives. More recently, two independent phase III studies showed that the MGMT promoter methylation status is of predictive value in the subpopulation of elderly glioblastoma patients [7, 8]. It was shown that patients with methylated MGMT promoter specifically benefited from temozolomide therapy, while patients with unmethylated MGMT promoter fared better when treated with radiotherapy alone. These results strongly argue for implementation of MGMT testing into everyday clinical patient care for informed patient allocation to radiotherapy or chemotherapy in order to improve patient outcomes, avoid unnecessary treatment toxicities and save costs. 

A number of different methods for analysis of MGMT testing have been reported, but many have been shown to be difficult to standardize. One of the most frequently used methods, methylation specific polymerase-chain reaction (MSP), has proven unreliable and is regarded by many laboratories as inadequately accurate for clinical decision making [9]. A fairly large number of modifications of this technique or alternative methods for MGMT testing have been proposed, but a consensus on a specific protocol reliably yielding high quality test results has not been reached so far [10]. 

In 2007, Mikeska et al. [11] optimized pyrosequencing (see Box A for methodological background) for testing of MGMT promoter methylation status in glioblastoma specimens and demonstrated feasibility and reliability in snap-frozen and formalin fixed paraffin-embedded specimens. Since then, several research groups have reported similarly favorable experience with this technique and demonstrated high reliability of test results and a better analytical performance as compared to other methods [11, 12, 13, 14, 15]. Furthermore, the MGMT status determined by pyrosequencing was shown to correlate with progression-free and overall survival of glioblastoma patients in several independent patient cohorts [12, 14, 16, 17]. Also the predictive value of the MGMT promoter methylation status seen in elderly glioblastoma patients in the NOA-08 and the Nordic glioma trials could be reproduced using pyrosequencing [7, 8, 18]. 

Herein, we assessed repeatability and reproducibility of MGMT testing by pyrosequencing among independent laboratories. 

**Box A.** Methodological background of pyrosequencing.

The pyrosequencing method is based on the “sequencing-by synthesis” principle and was developed at the Royal Institute of Technology (Stockholm, Sweden) between 1985 and 1996 [25, 26, 27, 28]. The technique relies on measurement of light signals (bioluminescence) that are generated by pyrophosphate release during DNA synthesis along a sequencing template with an annealed primer using specific enzymes (Klenow fragment of DNA polymerase I, ATP sulfurylase, luciferase, apyrase) and enzyme substrates (adenosine phosphosulfate, D-luciferin). The four nucleotides (C, T, G, A) are iteratively added in a cyclic reaction and the light signals emitted upon their incorporation into the newly synthesized complementary strand along the DNA template are recorded by a camera system. The nucleotide sequence of the DNA template can be deduced from the newly synthesized DNA sequence based on the Watson-Crick base pairing rule, according to which the nucleotide G will always pair with C and T will always pair with A. For analysis of DNA methylation, pyrosequencing is performed after prior bisulfite modification, which converts all unmethylated CpG dinucleotides to TpG, while methylated CpG dinucleotides remain unchanged [28].

## Study design 

### Tumor specimens 

For this study we used 18 glioblastoma specimens of 9 patients (1 formalin-fixed/paraffin-embedded (FFPE) and 1 RCL2-fixed/paraffin-embedded (RCLPE) sample per patient) [19]. The MGMT promoter methylation status has been determined for every included sample in a previous study using a methylation-sensitive restriction enzyme (MSRE)-based quantitative PCR (qPCR) assay in an independent laboratory (reference laboratory: Molecular Diagnostics, Austrian Institute of Technology). MGMT results obtained in the reference laboratory were validated by a MALDI-Epityper assay in another external laboratory as contract service (Sequenom, Hamburg, Germany) as described previously [20]. In brief, 5 patients were shown to harbor a methylated and 4 patients to harbor an unmethylated MGMT promoter in their tumor tissue. In each sample, the tumor cell content was more than 60% as determined on hematoxylin and eosin stained sections. Of each of the 18 tumor tissue blocks, we cut at least 30 µm material into an Eppendorf tube at the coordinating center (Institute of Neurology, Medical University of Vienna, Vienna, Austria) [20]. 

### Set-up of the ring trial 

**Figure 1:**
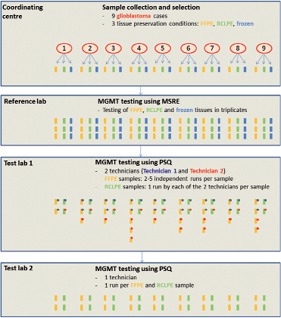
Cartoon showing the logistic and experimental set-up of this inter-laboratory study. FFPE = formalin-fixed and paraffin-embedded; MGMT = O6-methylguanine-methyltransferase promoter; MSRE = methylation-sensitive restriction enzyme-based polymerase chain reaction; PSQ = pyrosequencing; RCLPE = RCL2-fixed and paraffin-embedded.

The set-up of this interlaboratory study is summarized in Figure 1. 

The coordinating center sent the material to test laboratory 1 (Division of Pathology, Medical University of Innsbruck) and test laboratory 2 (Institute of Cancer Research, Medical University of Vienna). The investigators at test laboratories 1 and 2 were blinded to the MGMT status of the samples determined in the reference laboratory. 

In test laboratory 1, MGMT pyrosequencing of each FFPE tissue sample was performed at least twice and up to 5 times in independent runs and by two independent technicians (Table 1) (Figure 1). Of each RCLPE tissue sample, MGMT pyrosequencing was performed once by each of the two technicians. 

In test laboratory 2, MGMT pyrosequencing of each FFPE and each RCLPE tissue block was performed once by one technician (Table 1) (Figure 1). 

MGMT pyrosequencing results were reported back to the coordinating center by test laboratories 1 and 2 only after completion of all MGMT pyrosequencing analyses for un-blinding and comparison of test results. 

### DNA isolation, bisulfite modification, and pyrosequencing 

DNA isolation was performed using the EZ1 DNA investigator Kit (Qiagen, Germany, test laboratory 1) and the EpiTect FFPE Lysis Kit (Qiagen) (test laboratory 2) according to manufacturer’s recommendations. 

In both laboratories, bisulfite modification was performed using the Epi Tect Fast FFPE Bisulfite kit (Qiagen) according to the manufacturer’s recommendations. 

In both laboratories, analysis of the MGMT promoter methylation status was performed on a PyroMark Q24 (Qiagen, Germany) system and using the Therascreen MGMT Pyro Kit (Qiagen). 

MGMT-pyrosequencing yields a quantitative result giving the percentage of methylated alleles for each of the four investigated CpG sites (Figure 3). For definition of cases with methylated vs. unmethylated MGMT promoter, the percentage mean value of the methylation percentage obtained at each of the four investigated CpG dinucleotides was calculated. According to the definitions elaborated in a previous publication, cases with a mean methylation percentage of < 8% were regarded as MGMT promoter unmethylated and cases with a mean methylation percentage of ≥ 8% were regarded as MGMT promoter methylated [18]. 

## Results 

All 9 cases fulfilled the histopathological criteria of glioblastoma according to the current edition of the WHO Classification of Tumors of the Central Nervous System [21]. In each sample, the tumor cell content was more than 60% as determined on hematoxylin and eosin stained sections. Five patients were shown to harbor a methylated and four patients to harbor an unmethylated MGMT promoter in their tumor tissue. MGMT-pyrosequencing results of the individual runs are detailed in Table 1 and Table 2. We found a perfect correlation (100% concordance, Kappa value of 1, p < 0.001) of binary MGMT status (methylated vs. unmethylated) in all samples irrespective of tissue fixation conditions, testing laboratory, testing technician and time point of testing. MGMT promoter methylation status determined in test laboratories 1 and 2 were in perfect agreement with the results from reference laboratory. Thus, there was perfect reproducibility and repeatability of MGMT pyrosequencing. 

**Figure 2:**
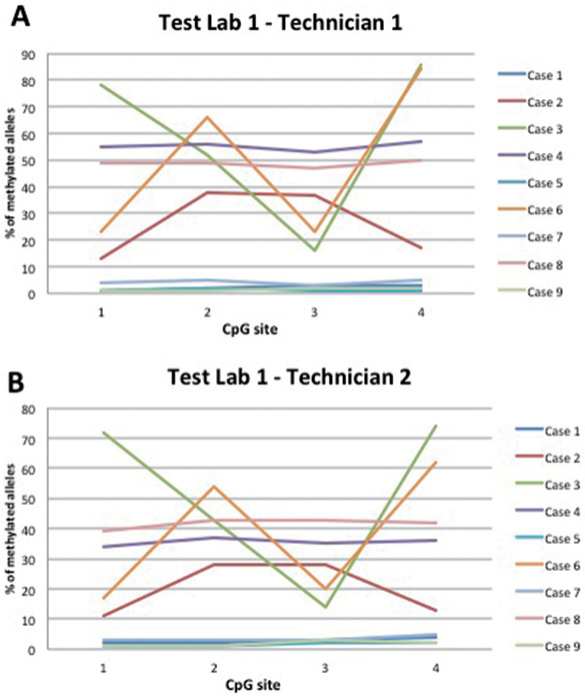
Percent (%) of methylated alleles for each of the investigated CpG sites. Experiment performed in test lab 1 by technician 1 (A) and technician 2 (B).

**Box B.** Clinical indications for MGMT promoter methylation testing.

MGMT promoter methylation testing by means of pyrosequencing fulfills the criteria of high clinical utility for predictive purposes in elderly glioblastoma and for prognostic purposes in all adult glioblastoma patients (Table 3).

## Discussion 

In this study, we show high reproducibility and repeatability of MGMT promoter methylation testing by means of pyrosequencing using a commercial assay. Our findings corroborate findings previously reported by other laboratories and confirm that pyrosequencing fulfills the prerequisites for MGMT testing in the routine clinical setting. We use in our laboratories the pyrosequencing device and kits commercialized by Qiagen, which have been proved to be easy to establish and use. It must be noted, however, that other pyrosequencing kits, both commercialized and non-commercialized, have been and are currently in use and may be equivalent alternatives for clinical applications, although conclusive comparative analyses are missing [11, 13, 15, 17, 18, 22, 23]. 

We feel that the confirmation of the high analytical performance of MGMT pyrosequencing is of major relevance for the care of glioblastoma patients, as it allows implementation of MGMT testing for prognostic and especially predictive purposes in the clinical setting. Based on the results of two recent phase III clinical trials and the confirmed assay reliability, MGMT pyrosequencing fulfills the criteria for high clinical utility and is recommended for therapy planning in elderly glioblastoma patients (Box B) (Table 3) [7, 8]. In other glioblastoma sub-cohorts, the clinical utility of MGMT testing is lower and at present does not have direct implications for clinical management [24], although knowledge of the MGMT promoter methylation status may support prognostic considerations that influence indirectly patient management. 

Although the evidence level for clinical MGMT testing is high, some issues remain to be addressed in further studies in order to refine its application. An important and not completely resolved issue is the definition of cut-offs for defining MGMT methylated vs. unmethylated cases based on the quantitative test results (the percentage of methylated alleles for each of the investigated CpG sites). For the assay used in our laboratories, an average methylation rate of the four analyzed CpG sites of 8% has been elaborated as clinically relevant cut-off in previous studies using MGMT pyrosequencing [12, 18]. Based on our data, this threshold seems reasonable, but it needs to be validated in prospective and adequately powered studies. Importantly, MGMT pyrosequencing provides quantitative results and there seems to be prognostic/predictive value of the continuous assay read-out. Dunn et al. [17] described that glioblastomas with the highest mean methylation levels (> 35%) showed the longest survival times. Similarly, Reifenberger et al. [18] reported that patients with strongly methylated tumors (> 25% MGMT methylated alleles) showed a significantly better outcome than patients with tumors with < 25% methylated alleles, if treated with alkylating chemotherapy. For the clarification and definition of universally applicable prognostic and predictive cut-off values, correlations of MGMT pyrosequencing results with patient outcomes in independent and large patient cohorts from prospective clinical trials are needed. 

**Figure 3.**
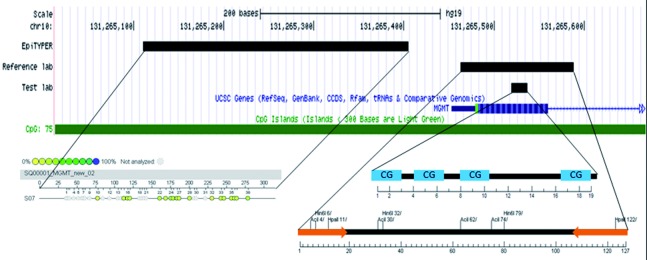
Overview of target sequences of the various assays used for O6-methylguanine-methyltransferase (MGMT) promoter methylation testing in our sample set. The reference laboratory used methylation-sensitive restriction enzyme-based polymerase chain reaction (MSRE) and the test laboratories used MGMT pyrosequencing.

Another important issue is the selection of appropriate CpG sites for methylation analysis. The MGMT gene contains 98 CpG sites in the first of five exons and the promoter regions (Figure 3). It is not entirely clear so far which of the CpG sites in the promoter region determine MGMT expression and have the greatest influence on the clinical course. The commercial kit we used in this study analyses four distinct CpG sites and a correlation with patient outcome has been shown for this particular assay [18]. It must be noted that implementation of alternative assays targeting other CpG sites will require to verify not only the analytical but also the clinical performance i.e. the prognostic/predictive impact. 

From a practical point of view, MGMT pyrosequencing is fraught with the disadvantage that it requires particular equipment and is relatively expensive. On the other hand, pyrosequencing performs very well not only on frozen but also on FFPE and RCLPE tissue samples. This facilitates centralization of MGMT testing in geographical regions, as paraffin-embedded samples can easily be transferred between institutions and pathology laboratories. In our opinion, however, regular accreditation of test laboratories through inter-laboratory ring trials seems vital to ensure high and sustained quality of MGMT testing. Quality assurance should also include histopathological verification of sufficient tumor content in the sample submitted for MGMT promoter methylation testing. 

In conclusion, we confirm in this study a high analytical performance of MGMT promoter methylation analysis by means of pyrosequencing (Table 3). Based on our results we can recommend this technique for clinical application in glioblastoma patients, given that strict quality controls including inter-laboratory ring trials are performed. 

## Acknowledgment 

We thank Anita Brandstetter for excellent technical assistance. 

## Conflict of interests 

The authors declare no conflict of interest. 

**Table 1.** Summary of the results of MGMT promoter methylation testing in cases with unmethylated MGMT promoter according to the reference laboratory. There is a perfect concordance) of MGMT status in all samples irrespective of tissue fixation conditions, testing laboratory, testing technician and time point of testing. Cut-offs for defining MGMT promoter methylated vs. unmethylated cases: for MSRE: 0.32%, for PSQ: 8%. PSQ-% represent the mean-methylation values from four CpG analyzed and calculated from pyrograms.

ID	Test lab	Test run	Fixation	Method	Technician	Mean value	Result
Case 1	Reference lab	1	RCLPE	MSRE	1	0	unmethylated
Case 1	Sequenom	2	RCLPE	Epityper	1	5.33	unmethylated
Case 1	Test lab 1	3	FFPE	PSQ	1	2.25	unmethylated
Case 1	Test lab 1	4	FFPE	PSQ	1	1.75	unmethylated
Case 1	Test lab 1	5	RCLPE	PSQ	1	2.25	unmethylated
Case 1	Test lab 1	6	RCLPE	PSQ	2	2.75	unmethylated
Case 1	Test lab 2	7	RCLPE	PSQ	1	2.27	unmethylated
Case 1	Test lab 2	8	FFPE	PSQ	1	2.55	unmethylated
Case 5	Reference lab	1	RCLPE	MSRE	1	0	unmethylated
Case 5	Sequenom	2	RCLPE	Epityper	1	3.9	unmethylated
Case 5	Test lab 1	3	FFPE	PSQ	1	1.75	unmethylated
Case 5	Test lab 1	4	FFPE	PSQ	1	2	unmethylated
Case 5	Test lab 1	5	FFPE	PSQ	2	1.75	unmethylated
Case 5	Test lab 1	6	RCLPE	PSQ	1	1.25	unmethylated
Case 5	Test lab 1	7	RCLPE	PSQ	2	1.5	unmethylated
Case 5	Test lab 2	8	FFPE	PSQ	1	2.29	unmethylated
Case 5	Test lab 2	9	RCLPE	PSQ	1	1.27	unmethylated
Case 7	Reference lab	1	RCLPE	MSRE	1	0	unmethylated
Case 7	Sequenom	2	RCLPE	Epityper	1	2.5	unmethylated
Case 7	Test lab 1	3	FFPE	PSQ	1	3.25	unmethylated
Case 7	Test lab 1	4	FFPE	PSQ	1	3	unmethylated
Case 7	Test lab 1	5	FFPE	PSQ	2	3.25	unmethylated
Case 7	Test lab 1	6	FFPE	PSQ	2	4	unmethylated
Case 7	Test lab 1	7	RCLPE	PSQ	1	4.25	unmethylated
Case 7	Test lab 1	8	RCLPE	PSQ	2	3.5	unmethylated
Case 7	Test lab 2	9	RCLPE	PSQ	1	2.48	unmethylated
Case 7	Test lab 2	10	FFPE	PSQ	1	2.81	unmethylated
Case 9	Reference lab	1	RCLPE	MSRE	1	0.02	unmethylated
Case 9	Sequenom	2	RCLPE	Epityper	1	1.9	unmethylated
Case 9	Test lab 1	3	FFPE	PSQ	1	5	unmethylated
Case 9	Test lab 1	4	FFPE	PSQ	1	5.25	unmethylated
Case 9	Test lab 1	5	FFPE	PSQ	2	5.25	unmethylated
Case 9	Test lab 1	6	FFPE	PSQ	2	5.75	unmethylated
Case 9	Test lab 1	7	RCLPE	PSQ	1	1.5	unmethylated
Case 9	Test lab 1	8	RCLPE	PSQ	2	1.75	unmethylated
Case 9	Test lab 2	9	RCLPE	PSQ	1	1.06	unmethylated
Case 9	Test lab 2	10	FFPE	PSQ	1	3.84	unmethylated

FFPE = formalin-fixed and paraffin-embedded; MSRE = methylation-specific restriction enzyme based quantitative polymerase chain reaction; PSQ = pyrosequencing; RCLPE = RCL2-fixed and paraffin-embedded.

**Table 2.** Summary of the results of MGMT promoter methylation testing in cases with methylated MGMT promoter according to the reference laboratory. There is a perfect correlation (100% concordance) of MGMT status in all samples irrespective of tissue fixation conditions, testing laboratory, testing technician and time point of testing. Cut-offs and abbreviations: same as Table 1.

ID	Test lab	Test run	Fixation	Method	Technician	Mean value	Result
Case 2	Reference lab	1	RCLPE	MSRE	1	0.56	methylated
Case 2	Sequenom	2	RCLPE	Epityper	1	16.9	methylated
Case 2	Test lab 1	3	FFPE	PSQ	1	21.25	methylated
Case 2	Test lab 1	4	FFPE	PSQ	1	21.75	methylated
Case 2	Test lab 1	5	FFPE	PSQ	2	20.50	methylated
Case 2	Test lab 1	6	RCLPE	PSQ	1	26.25	methylated
Case 2	Test lab 1	7	RCLPE	PSQ	2	20	methylated
Case 2	Test lab 2	8	FFPE	PSQ	1	31.35	methylated
Case 2	Test lab 2	9	RCLPE	PSQ	1	25.64	methylated
Case 3	Reference lab	1	RCLPE	MSRE	1	0.69	methylated
Case 3	Sequenom	2	RCLPE	Epityper	1	20.9	methylated
Case 3	Test lab 1	3	FFPE	PSQ	1	28.5	methylated
Case 3	Test lab 1	4	FFPE	PSQ	1	30.25	methylated
Case 3	Test lab 1	5	FFPE	PSQ	2	34.75	methylated
Case 3	Test lab 1	6	RCLPE	PSQ	1	58	methylated
Case 3	Test lab 1	7	RCLPE	PSQ	2	50.75	methylated
Case 3	Test lab 2	8	FFPE	PSQ	1	36.21	methylated
Case 3	Test lab 2	9	RCLPE	PSQ	1	48.94	methylated
Case 4	Reference lab	1	RCLPE	MSRE	1	7.35	methylated
Case 4	Sequenom	2	RCLPE	Epityper	1	15	methylated
Case 4	Test lab 1	3	FFPE	PSQ	1	17.75	methylated
Case 4	Test lab 1	4	FFPE	PSQ	1	18.25	methylated
Case 4	Test lab 1	5	FFPE	PSQ	2	18.75	methylated
Case 4	Test lab 1	6	FFPE	PSQ	2	16.5	methylated
Case 4	Test lab 1	7	FFPE	PSQ	2	18.5	methylated
Case 4	Test lab 1	8	RCLPE	PSQ	1	55.25	methylated
Case 4	Test lab 1	9	RCLPE	PSQ	2	35.5	methylated
Case 4	Test lab 2	10	FFPE	PSQ	1	18.03	methylated
Case 4	Test lab 2	11	RCLPE	PSQ	1	34.94	methylated
Case 6	Reference lab	1	RCLPE	MSRE	1	11.85	methylated
Case 6	Sequenom	2	RCLPE	Epityper	1	23.4	methylated
Case 6	Test lab 1	3	FFPE	PSQ	1	20.25	methylated
Case 6	Test lab 1	4	FFPE	PSQ	1	20.5	methylated
Case 6	Test lab 1	5	FFPE	PSQ	2	15.25	methylated
Case 6	Test lab 1	6	FFPE	PSQ	2	14.5	methylated
Case 6	Test lab 1	7	RCLPE	PSQ	1	49	methylated
Case 6	Test lab 1	8	RCLPE	PSQ	2	38.25	methylated
Case 6	Test lab 2	9	FFPE	PSQ	1	24.42	methylated
Case 6	Test lab 2	10	RCLPE	PSQ	1	36.23	methylated
Case 8	Reference lab	1	RCLPE	MSRE	1	69.38	methylated
Case 8	Sequenom	2	RCLPE	Epityper	1	31.3	methylated
Case 8	Test lab 1	3	FFPE	PSQ	1	30.25	methylated
Case 8	Test lab 1	4	FFPE	PSQ	1	31.25	methylated
Case 8	Test lab 1	5	FFPE	PSQ	2	40.25	methylated
Case 8	Test lab 1	6	RCLPE	PSQ	1	48.75	methylated
Case 8	Test lab 1	7	RCLPE	PSQ	2	41.75	methylated
Case 8	Test lab 2	8	RCLPE	PSQ	1	37.75	methylated
Case 8	Test lab 2	9	FFPE	PSQ	1	33.98	methylated

**Table 3.** Levels of evidence for MGMT promoter methylation testing using various DNA-based methods. Definitions and discussion of evidence levels A, B, C and D are given in [24]; in brief, a biomarker needs to reach evidence level B or higher for both analytical and prognostic or predictive performance to have sufficient clinical utility.

Biomarker	Tumor type	Analytical performance	Prognostic clinical performance	Predictive clinical performance
*MGMT* promoter methylation	Adult glioblastoma	A: PSQ	A: glioblastoma	A: glioblastoma of the elderly
		B:	B:	B:
		C: MSP, MS-MLPA, MS-PCR	C:	C: glioblastoma (all adult patients except elderly)
		D:	D:	D:

MGMT = O6-methylguanine-methyltransferase promoter; MSP = methylation-specific polymerase chain reaction; MS-MLPA = methylation-specific multiplex ligation-dependent probe amplification; PSQ = pyrosequencing.
